# Supplementary material for: Are carbon emissions trading and green financial instruments synergistic? -Comprehensive quantitative research based on content analysis
Source: PLoS One. 2024 Mar 7;19(3):e0298601. doi: 10.1371/journal.pone.0298601 (PMC10919601; doi:10.1371/journal.pone.0298601)
Supplement: S1 Data — (ZIP) [file pone.0298601.s001.zip › Data/地级市Z_F工作报告.zip!/╔╜╢1⁄2/╟α╡║╩╨/└┤╫╘╣1⁄2╓┌║┼í╛┬φ┐╦╩2╛▌═°í┐_2016.docx]

各位代表：

　　现在，我代表市人民政府向大会报告工作，请予审议，并请各位政协委员和其他列席人员提出意见。

　　一、“十二五”时期工作回顾

　　“十二五”时期是青岛发展很不平凡的五年。全球经济低迷，国内经济也处于发展速度换挡期、结构调整阵痛期、前期刺激政策消化期。面对困难和挑战，全市人民团结一心，深入贯彻党的十八大和十八届三中、四中、五中全会精神，全面落实习近平总书记系列重要讲话和视察山东重要讲话、重要批示精神，认真落实市第十一次党代会提出的目标任务，主动适应经济发展新常态，坚持世界眼光、国际标准，发挥本土优势，深入推进蓝色引领、全域统筹、创新驱动发展战略，积极寻标对标达标夺标创标，努力加速提升创新增效落实，在建设宜居幸福的现代化国际城市进程中取得重大成就。

　　五年来，全市生产总值跨越四个千亿元台阶，从5666亿元增加到9300亿元，年均增长9.7%，人均生产总值超过1.6万美元；一般公共预算收入跨越六个百亿元台阶，从453亿元增加到1006亿元，年均增长17.3%；固定资产投资累计完成2.5万亿元，年均增长19.4%；社会消费品零售总额从1961亿元增加到3714亿元，年均增长13.6%；城乡居民人均可支配收入分别从24380元、9326元提高到40370元、16730元，年均分别增长10.6%、12.4%，城乡收入比从2.61缩小到2.41；城镇登记失业率控制在4%以内。生产总值健康度位居副省级城市第一位，综合竞争力全面提升。

　　五年来，坚持转型升级、提质增效，产业结构调整迈出新步伐。突出蓝色高端新兴导向，加大腾笼换鸟、凤凰涅槃力度，着力打造经济升级版。实施蓝色跨越三年行动计划，海洋生物医药、海洋新材料、海洋工程等产业蓬勃发展，海洋生产总值达到2093.4亿元，占生产总值的22.5%。十条工业千亿级产业链产值占规模以上工业总产值的75%。十个服务业千万平米工程建成运营6169万平方米。金融业增加值达到588亿元，年均增长16.3%。旅游业总收入达到1199亿元，年均增长15.6%。服务业占生产总值的52.8%，提高6.4个百分点。现代农业十大重点工程加快推进，粮食连年丰收，现代畜牧养殖业健康发展，远洋渔业产量从3000吨增加到14万吨，增幅居全国第一。成为国家现代农业示范区、全国农业农村信息化示范基地。战略性新兴产业占规模以上工业总产值的20%。394个市级重点建设项目竣工，一汽大众华东生产基地、惠普全球大数据中心、万达东方影都、中铁世界博览城等一批大项目加快推进。青钢集团、青岛碱业等老厂区关停，72户企业搬迁改造竣工，老城区企业环保搬迁取得决定性成果。

　　五年来，坚持创新驱动、人才优先，创新发展取得新突破。把创新摆在发展全局的核心位置，努力建设创新之城、创业之都、创客之岛。全社会研发经费投入占生产总值比重从2.2%上升到2.81%；市级以上重点实验室、企业技术中心、工程技术研究中心达到908家；高新技术企业达到964家，新增675家。海洋科学与技术国家实验室、国家深海基地投入使用，中科院、中船重工、中国电科、中海油、机械总院等17个国字号科研产业基地建成运营，西安交大、哈工程、天津大学、清华大学等10所重点高校先后在青建立研发机构，阿斯图中俄科技园、中乌特种船舶研究设计院等8个国际高端研发平台加快建设，科研实力和创新能力再上新台阶。完善创新服务体系，千万平米孵化器工程累计开工1153万平方米，竣工1001万平方米，入驻企业超过5000家；千万平米软件产业园累计开工475万平方米，竣工380万平方米，软件业务收入年均增长42.7%；千万平米人才公寓开工450万平方米，竣工185万平方米。累计发明专利授权1.26万件，是上个五年的5.5倍。技术合同交易额达到89.5亿元，是2010年的5.5倍。获批建设国家海洋技术转移中心。大力实施百万人才集聚行动，引进、培养各类人才51.5万人。创新科技资金投入机制，组建11只天使投资基金，引导社会资本投入创新创业。获批国家创新型试点城市、科技金融结合试点城市、知识产权示范城市和科技服务业区域试点城市。

　　五年来，坚持区域协同、城乡一体，全域统筹呈现新格局。深入实施全域统筹、三城联动、轴带展开、生态间隔、组团发展的城市空间发展战略，空间布局趋于科学合理，发展方向和功能定位趋于清晰明确，组团式、生态化的海湾型大都市建设格局全面展开。新一轮城市总体规划获国务院批复，青岛由过去定位的中国东部沿海重要的经济中心城市，提升定位为国家沿海重要中心城市和滨海度假旅游城市、国际性港口城市、国家历史文化名城。依据总体规划，加快审批进度，助推经济社会发展，批复规划许可面积累计1.56亿平方米，是上个五年的1.49倍。“一谷两区”等重点功能区加快建设，引领和带动作用明显增强。蓝谷核心区获批国家科技兴海产业示范基地，12个重大科研平台加快建设，对建设海洋强市产生重大推动作用。西海岸新区获批第九个国家级新区，全面推进改革创新和转型发展，国家政策试点全面实施，产业升级步伐加快，成为带动全市发展的增长极。红岛经济区高新技术主导产业快速发展，科技生态人文新城初具规模。突破发展市北、李沧和平度、莱西，城乡差距、南北差距加快缩小。市南总部经济中心区、市北中央商务区、李沧交通商务区、崂山金家岭财富金融聚集区、城阳轨道交通产业区等加快建设。即墨汽车产业基地、胶州临空经济区、平度南村家电产业园、莱西新能源汽车产业园等区域特色产业不断壮大，四市财政收入实现翻番以上增长。新型城镇化进程加快，小城市培育试点和省级示范镇建设扎实推进。新型农村社区累计开工408个，建成回迁217个，32万农民搬进新居。累计建成新型农村社区服务中心1057个，农村社区化服务覆盖率达到90%以上。常住人口城镇化率达到69.99%，提高4.18个百分点。

　　基础设施建设实现重大突破。胶州湾隧道、胶州湾大桥、铁路青岛北客站建成使用，龙青高速公路、青荣城际铁路建成通车，济青高铁、青连铁路开工建设。重庆路改造、崂山路改造、金水路拓宽等工程顺利完成。城市道路和排水管网分别增长27.1%、30%。市区集中供热普及率从62%提高到82%。天然气供应量、电网配电容量均实现翻番。邮轮母港客运中心启用。港口吞吐量和集装箱吞吐量分别增长39.3%、45.2%。完成机场新一轮布局调整，新机场全面开工建设，空港新增9条国际航线，旅客吞吐量增长64%，青岛与世界的联系更加畅通快捷。城市轨道交通在建230公里，3号线北段开通运营，青岛迈入地铁时代。

　　五年来，坚持深化改革、扩大开放，经济社会发展焕发新活力。聚焦中央和省委部署，聚焦重点功能区，聚焦瓶颈难题，聚焦民生改善，重要领域和关键环节改革取得突破。完成新一轮政府机构改革，稳妥推进事业单位分类改革，县域行政综合执法改革试点取得明显成效。公布实施市区两级行政权力清单和责任清单，行政审批事项精简55%，前置审批事项减少93%。大力推进并联审批、网上审批、一站式办理，群众办事更加便捷。初步建立统一的公共资源交易平台。创新财政专项资金运作方式，释放社会投资活力，民间投资比重达到75.7%。深化国企改革，市直企业所属企业80%以上实现混合所有制，国有资产增长1.5倍。推进商事制度改革，加大对中小微企业的政策支持力度，市场主体达到95.3万户，增长1.2倍。财富管理金融综合改革试验区建设取得实质性进展，首批财富管理创新试点政策顺利实施。法人金融机构和外资金融机构分别达到22家、34家，均实现翻番。境内外上市企业增加18家，新三板和蓝海股权交易中心挂牌企业分别达到57家、318家。出台了居民用水、用气阶梯价格制度。推进户籍制度改革，在全省率先实施积分落户政策。加快农村土地“三权”分置改革，基本完成土地确权登记颁证。完成837个村集体产权制度改革。推进农村“四权”抵押贷款，金融支农实现新突破。培育新型职业农民8.5万人，新型农业经营主体发展到1.5万个。

　　对外开放水平持续提升。积极融入“一带一路”战略，成为新亚欧大陆桥经济走廊主要节点城市和海上合作战略支点，拥有6个国家级经济园区、4个海关特殊监管区，在全国开放大局中的地位更加凸显。年实际利用外资跨越四个10亿美元台阶，累计达到265.3亿美元，是上个五年的1.8倍。累计货物进出口3725亿美元，是上个五年的1.6倍。服务外包离岸执行额实现倍增。对外承包工程营业额增长3.5倍。保税港区汽车整车进口口岸建成运营，成为全国第二大平行车进口口岸。电子口岸平台加快建设，口岸通关效率不断提升。东亚海洋合作平台永久性会址落户青岛。中德生态园成为中德经济合作重要平台。我市成为首批中美地方经贸合作试点城市、中英地方投资贸易合作重点城市。成功举办两轮中美投资协定谈判活动、首次中日韩自贸区副部长级谈判活动、亚太经合组织贸易部长会议和第二次高官会、首届国际教育信息化大会。国际海事司法研究基地和国家法官学院青岛海事分院落户。设立4处境外青岛工商中心。新增4个友好城市、9个友好合作关系城市、30个国际经济合作伙伴关系城市。连续四年入选“魅力中国—外籍人才眼中最具吸引力的城市”。扩大国内经济合作，累计利用内资8668亿元，对口支援工作扎实推进。

　　五年来，坚持生态环保、绿色发展，美丽青岛建设再上新水平。深入实施生态市创建行动计划，加快城市绿道建设，建成30处万亩林场，新增造林73.2万亩，林木绿化率达到40%，获得国家森林城市称号。成功举办世界园艺博览会，并获得鲁班奖和詹天佑奖，成为园林艺术和城市生态建设的优秀范例。严格环保执法，推行清洁生产，加强大气和水污染治理，连续三年获得省污染减排考核一等奖。严格生态保护红线制度，建立完善生态补偿和保护长效机制。全部淘汰高污染黄标车10.8万辆，更新节能环保型公交车2822辆，全市禁止新上传统燃煤项目，完成469个燃煤锅炉废气治理项目，市区空气质量得到改善。加强胶州湾海洋生态综合整治，优良水域面积比例达到65%，提高7.8个百分点。成为国家级海洋生态文明建设示范区。全面实施河流污染整治，省控重点河流水质达到改善标准。新建改建污水处理厂18处，市区污水集中处理率达到98%，提高11个百分点。大沽河全线127公里综合整治工程全面竣工，展现出洪畅、堤固、水清、岸绿、景美的母亲河新形象，改善了流域生态环境，促进了区域经济文化发展和美丽乡村建设，惠及到沿岸2513个村庄、240万人。

　　五年来，坚持以人为本、保障民生，群众生活得到新改善。全市民生支出累计投入3315亿元，年均增长22%。实施城乡就业一体化政策，建立了劳动力资源管理信息系统，实行重点建设项目与就业培训联动。推进大众创业工程，成为全国创业先进城市。全面实施城乡居民养老保险制度，城镇职工医疗保险、城镇居民医疗保险、新型农村合作医疗实现“三险合一”。企业退休人员基本养老金达到2914元，提高64.9%；城乡低保标准分别达到每人每月620元、420元，分别提高77%、180%；城乡困难居民医疗救助标准实现均等化。实施精准扶贫，10万贫困人口实现脱贫。千名老人养老床位从19.8张增加到37张，政府为困难老人购买居家养老服务实现全覆盖。建立以公共租赁住房为主体的住房保障和供应体系，开工建设保障性住房9.6万套，基本建成5.8万套。改造棚户区8万户、农村危房2.8万户，受益居民30多万人。

　　教育卫生事业实现新发展。新建改建幼儿园969所，公办和普惠性民办幼儿园比重达到73.4%。投资172亿元，新建改建中小学729所，分别是上个五年的4倍和2.1倍，87.8%的普通中小学达到市现代化学校标准。学前教育普及水平、义务教育校际均衡水平在副省级城市中位居第一。义务教育、中职教育和特殊教育实现免费。山东大学青岛校区部分建成，北京外国语大学、上海财经大学、对外经贸大学等院校已来青办学。投资82亿元，建设医疗卫生设施31处，建筑面积122万平方米，是上个五年的1.5倍。妇女儿童医院、齐鲁医院青岛院区、市第三人民医院等项目建成使用，新增医疗床位1.2万张。深入推进县级公立医院综合改革，全面实施基本药物制度，不断提升基层医疗机构服务水平，居民医保个人负担率明显下降。人均预期寿命提高0.9岁，达到81岁。

　　重点领域治理得到加强。严格落实安全生产责任，加强安全隐患排查治理，安全生产基础工作持续加强。优化交通组织，完善交通安全设施，交通智能化管理水平稳步提升。突出公交优先，市区新增公交线路138条。加强食品药品安全整治，强化农业面源污染防治，确保人民群众“舌尖上的安全”。实施消费市场秩序专项治理，严厉打击各类违法行为。连续五年开展城乡环境综合整治行动，顺利通过国家卫生城市复审。开展乡村文明行动，实现城乡环卫一体化。城市应急处置能力和应急管理水平进一步提升。深化信访工作制度改革，引导群众依法理性反映诉求。创新立体化社会治安防控体系，社会治安环境持续优化。

　　五年来，坚持文化强市、文化惠民，城市软实力实现新提升。加强思想道德建设，弘扬中华优秀传统文化，中国梦和社会主义核心价值观深入人心。大力开展群众性精神文明创建活动，涌现出一批先进典型，“微尘”等成为全国知名爱心品牌，蝉联全国文明城市荣誉称号。群众性文化活动蓬勃开展，覆盖城乡的四级公共文化服务网络基本形成，成为首批国家公共文化服务体系示范区。4部作品获得“五个一工程”奖，舞剧《红高粱》获得文华大奖。圆满举办东亚文化之都系列活动。文化体制改革成效显著，城市传媒集团成为全省首家文化上市企业。获批全国版权示范城市。灵山湾文化产业区加快建设。文化创意产业成为支柱产业。连续举办全国青少年小提琴比赛、世界杯帆船赛青岛站等活动，荣获世界帆船运动突出贡献奖。成功举办世界休闲体育大会。青岛籍运动员在国内外大型体育赛事上取得优异成绩。圆满完成2012中俄海上联合军演、西太平洋海军论坛等重大军事活动保障工作。拥军优属、军民共建深入开展。人防建设与经济社会融合发展。气象、防震减灾工作有序开展。侨务、对台和民族宗教工作得到加强。仲裁、司法作用进一步发挥。统计、档案、史志工作成效明显。老龄、慈善、残疾人事业不断进步。妇女儿童、青少年事业健康发展。

　　五年来，坚持依法行政、廉洁从政，民主法治建设实现新进步。坚决执行人大及其常委会的决议、决定，坚持重大事项向人大报告、向政协通报制度，自觉接受人大法律监督、工作监督和政协民主监督。提请市人大常委会审议法规草案25件，新制定政府规章33件，修订、废止政府规章11件。认真办复市人大代表建议2906件、市政协提案3480件，推动了胶州湾保护、农业面源污染等突出问题的有效解决。出台重大行政决策程序规定，科学民主依法决策机制进一步健全。成立行政复议委员会。健全行政裁量权基准制度，建立行政执法全过程记录制度，提高了行政执法规范化水平。推进政务信息全面公开，建成网上便民服务大厅和政府数据开放网站。积极回应群众关切，每年开展“三民”活动，网络在线问政、行风在线、政务服务热线等常态化政民互动渠道更加畅通，青岛政务网在全国政府网站绩效评估中连续三次位居第一。扎实开展党的群众路线教育实践活动和“三严三实”专题教育，制定实施鼓励改革创新治理庸政懒政的意见，服务效能明显提升。严格落实领导干部“一岗双责”，出台进一步加强政府廉政建设的实施意见。严格执行厉行节约规定，市级“三公”经费预算逐年下降。坚决查处“四风”、失职渎职和腐败问题，纪检监察机关累计立查案件4258件，党纪政纪处分4126人。在市委坚决贯彻中央决策部署、坚定有力的落实推进下，反腐力度和法纪规矩全面强化，政治生态明显净化，干部作风明显变化，从政环境明显优化，全市上下凝心聚力、干事创业的正能量彰显。

　　各位代表，去年，全市稳增长、促改革、调结构、惠民生、防风险也取得较好成效，继续保持了经济稳中有进、民生持续改善的良好态势。全市生产总值增长8.1%，一般公共预算收入增长12.4%，城乡居民人均可支配收入分别增长8.1%、8.4%，城镇登记失业率控制在3%，居民消费价格上涨1.2%，人口自然增长率2‰，全面完成了节能减排各项任务。

　　经济实现平稳增长。坚持运行要畅、投资要顺、转型要快、调节要灵，持续开展调结构稳增长抓落实现场推进活动，走访千家企业、走进千个项目现场、走谈千个项目，及时出台和落实稳增长政策。减免企业税费，降低工商业电价，减轻企业负担134亿元。200个市级重点建设项目全部开工，完成投资1503亿元。固定资产投资达到6556亿元，增长14.2%。休闲、文体、信息等消费快速增长，社会消费品零售总额增长10.5%。青岛进入中国大陆最佳商业城市前十强。外贸出口实现正增长，对外贸易总额突破800亿美元。实际利用外资达到67亿美元，居副省级城市前列。

　　发展动力不断增强。出台创新创业扶持政策，推进大众创业、万众创新，26家众创空间纳入国家级孵化器体系。科技型中小企业加快发展，“千帆计划”企业达到1573家。实施“互联网+”发展规划，成功举办首届世界互联网工业大会。物联网、机器人、三维（3D）打印、石墨烯材料、新能源汽车等新兴产业呈现良好发展势头。实施“海洋+”发展规划，海洋生产总值增长15.1%。驰名商标达到123件，成为全国质量强市示范城市。

　　改革开放纵深推进。各领域改革不断深化，完成14项中央、省级改革试点任务。国内首家产融结合消费金融公司、第三只人民币投贷基金落户，青岛国富金融资产交易中心、青岛场外市场清算中心成立。成为国内贸易流通体制改革发展综合试点城市、国家旅游业改革创新先行区。胶州获批全国综合行政执法体制改革试点。平度国家中小城市综合改革试点扎实推进。积极复制推广上海等自贸试验区经验，42项改革试点成效明显。出台落实“一带一路”战略规划总体实施方案。以青岛为龙头，实施丝绸之路经济带海关、检验检疫区域一体化通关改革。获批国家跨境电子商务综合试验区。服务进出口突破100亿美元。对外投资33亿美元，增长124.2%。

　　民生保障水平持续提升。提高19项民生保障标准，扩大2项民生保障范围。11件市办实事全部完成。新增城乡就业41.4万人，政策扶持创业2.3万人。建设中小学标准化食堂212所。建成189家医联体，新建22家社区卫生服务中心（站），在岗乡村医生待遇提高30%。新增社区老年人日间照料中心215个。长期医疗护理保险制度荣获政府创新最高奖。为13.6万名低保对象发放低保金8.3亿元，为3.6万名困难群众发放医疗救助金1.7亿元，为1.68万户困难群众发放临时救助金4542万元。完成农村规模化供水工程27处，规模化供水人口覆盖率达到95%。新疆路高架快速路、福州路打通、株洲路打通等工程主线通车，完成91条道路大中修、13处道路微循环改造，完善交通设施1100余处，新增公共停车泊位5500个。市区空气质量优良率达到80.3%，提高8.5个百分点。

　　各位代表，2015年主要目标任务的完成标志着“十二五”规划圆满收官。五年的实践使我们深刻认识到，做好政府工作，必须始终坚定不移地贯彻落实党中央国务院、省委省政府的决策部署，在市委的坚强领导下，紧密结合实际，创造性地开展工作；必须始终坚持人民主体地位，尊重基层首创精神，充分发挥社会各界的积极性、主动性和创造性；必须始终保持强烈的危机感和责任感，牢牢把握大局，善于抢抓机遇，勇于攻坚克难，奋力争创一流；必须不断强化法治观念、法律意识，善于运用法治思维和法治方式破解难题，使政府工作始终在法治轨道上运行；必须统筹谋划、协调推进，突出补短板、促均衡、强基础、增后劲，努力实现科学发展、可持续发展和包容性发展。五年来，全市广大干部履职尽责、积极进取，市人大、市政协和社会各界大力支持、加强监督，全市上下、各行各业同心协力、拼搏奉献，每一位建设者都为我们这座城市的发展贡献了智慧与力量。在此，我代表青岛市人民政府，向全体市民和来青建设者，向人大代表和政协委员，向中央、省驻青单位，向驻青部队和武警官兵，向社会各界人士和离退休老同志，向所有关心支持青岛发展的海内外朋友们，表示衷心的感谢和崇高的敬意!

　　各位代表，青岛的发展还面临着不少困难和挑战，政府自身工作还存在许多不足和差距。稳增长压力较大，新旧动力转换任务艰巨。高层次、高技能人才不足，新产业、新业态发展不快，自主创新能力不强。现代服务业水平不够高，制造业转型升级亟待加速。财政减收增支因素多，金融环境仍需改善。解决城乡、区域发展不协调问题尚需进一步突破。城市卫生管理薄弱环节多，雾霾和交通拥堵治理任务重，城市环境、秩序和服务都有待改善，依法治市需进一步强化。就业、保障、住房、交通、教育、医疗、养老、食品安全、安全生产等民生工作还存在不少短板。有的领导干部担当意识不强，有些工作人员作风不严不实问题仍然存在，法治意识和法治政府建设有待强化，一些领域的消极腐败现象时有发生。对此，我们一定高度重视，接受社会监督，尽心尽力尽责地努力解决。

　　二、“十三五”时期目标任务

　　今后五年，是青岛率先全面建成较高水平小康社会的决胜阶段，也是加快建设宜居幸福现代化国际城市的关键时期。十八届五中全会为我们进一步适应、把握、引领新常态提供了行动指南，市委十一届十次全会确定了我市“十三五”发展的目标任务。我们必须准确把握战略机遇期内涵的深刻变化，充分利用“十二五”发展创造的有利条件，更加有效地应对各种风险和挑战，尚实干、勇作为、敢担当，突出新、追求优、敢争先，不断开拓发展新境界，努力实现更高质量和效益、更加公平和可持续的新发展。

　　“十三五”时期，要高举中国特色社会主义伟大旗帜，全面贯彻党的十八大和十八届三中、四中、五中全会精神，以马克思列宁主义、毛泽东思想、邓小平理论、“三个代表”重要思想、科学发展观为指导，深入贯彻习近平总书记系列重要讲话和视察山东重要讲话、重要批示精神，按照“五位一体”总体布局和“四个全面”战略布局，坚持发展是第一要务，坚持创新、协调、绿色、开放、共享发展理念，按照省委“一个定位、三个提升”要求，坚持世界眼光、国际标准，发挥本土优势，以提高发展质量和效益为中心，加快形成引领经济发展新常态的体制机制和发展方式，破解发展难题、增强发展动力、厚植发展优势，率先全面建成较高水平小康社会，进而向基本实现现代化目标迈进，努力谱写中国梦青岛新篇章。

　　今后五年发展的主要目标是：

　　——经济保持中高速增长。在强化平衡性、包容性、可持续性发展的基础上，提前实现经济总量和城乡居民人均可支配收入比2010年翻一番，发展质量和效益明显提升。全市生产总值年均增长7.5%左右，城乡居民人均可支配收入增长7.5%以上。产业迈向中高端水平，信息化和工业化深度融合，服务业比重达到57%，农业现代化水平明显提高。全社会研发经费占比提高到3.2%，创新能力和科技进步贡献率大幅提升。加快打造国内重要的互联网工业强市和智能制造基地，建设国家东部沿海重要的创新中心、国内重要的区域性服务中心、国际先进的海洋发展中心，充分发挥国家“一带一路”战略节点和支点功能、东北亚航运枢纽功能，基本建成具有国际影响力的区域性经济中心城市。

　　——发展协调性全面增强。区域协调、城乡统筹发展体制机制不断完善，全域空间布局更加优化，三个中心城区、十个次中心城区和镇（街道）村（社区）协同发展，功能互补、特色鲜明、优美宜居的现代城乡形态基本形成。以人为核心的新型城镇化加快推进，常住和户籍人口城镇化率分别提高到72%、60%。全域南北差距、城乡差距大幅缩小。军民深度融合共创共建取得明显成效。

　　——人民生活品质普遍提高。就业比较充分，创业带动就业取得明显成效。基本公共服务水平和均等化程度稳步提高，劳动年龄人口平均受教育年限明显增加。中等收入人口比重上升，提前完成市定标准贫困人口脱贫攻坚任务，全面完成城区棚户区和危房改造任务。社会保障体系更加健全，市民健康水平明显提升，平安青岛建设取得新进展，群众对改革发展的获得感幸福感明显增强。

　　——市民素质和社会文明程度实现新提升。中国梦和社会主义核心价值观更加深入人心，爱国主义、集体主义、社会主义思想广泛弘扬。城市精神内涵得到深度挖掘和丰富，凝聚力、创造力和影响力明显提升。文明创建活动向纵深发展，市民思想道德、科学文化和健康素质明显提高，全社会法治意识不断增强。文化事业繁荣发展，公共文化服务体系更加完善。基本公共教育均衡协调发展，终身教育体系更加完善。

　　——生态环境质量显著改善。低碳绿色生产方式和生活理念不断巩固，生态文明制度基本健全。节能减排降耗的长效机制逐步完善，全面完成国家下达的约束性目标。生态市建设扎实推进，主体功能区布局和生态安全屏障基本形成，人居生态环境建设走在全国前列，建设宜居幸福的美丽城市。

　　——城市治理体系和治理能力现代化取得重大进展。体制机制创新实现新突破，重要领域和关键环节改革取得决定性成果。城市管理更加科学高效，城市国际品质和宜居水平明显提升。人民民主更加健全，依法治市加快推进，法治政府基本建成，司法公信力明显提高。人民群众权益得到切实保障，产权得到有效保护。开放型经济新体制新格局更加完善。基层社会治理体系更加健全。

　　我们坚信，有中央和省、市委的坚强领导，有长期发展奠定的坚实基础，有全市人民的不懈奋斗，青岛一定能够在新常态发展中实现超越，全面完成“十三五”奋斗目标，率先走在前列。

　　三、2016年主要工作安排

　　2016年是实施“十三五”规划开局之年，也是本届政府收官之年。做好今年工作，必须主动适应经济发展新常态，坚持改革开放，坚持稳中求进，坚持稳增长、调结构、惠民生、防风险，加强结构性改革，抓好去产能、去库存、去杠杆、降成本、补短板工作，加快培育新的发展动能，改造提升传统比较优势，增强持续增长动力，推动各项工作实现新提升、迈出新步伐，努力实现“十三五”时期经济社会发展的良好开局。主要预期目标是：全市生产总值增长7.5%－8%，一般公共预算收入增长9%左右，固定资产投资增长12%左右，社会消费品零售总额增长11%左右，对外贸易总额增长2.5%左右，城乡居民人均可支配收入分别增长8%和8.5%左右，城镇登记失业率控制在4%以内，居民消费价格涨幅控制在3%以内，完成年度节能减排任务。重点做好以下工作：

　　（一）坚持创新发展，着力提高发展质量和效益。始终把发展基点放在创新上，更多依靠创新驱动，更好发挥本土优势，在建设创新型城市上率先走在前列。

　　推进供给侧结构性改革。抓好经济运行，确保在调整中实现稳增长目标。多措并举化解过剩产能，重点妥善处置工业“僵尸企业”、房地产“僵尸项目”等问题，加大兼并重组力度，规范企业破产，实现市场出清。以满足新市民住房需求为主要出发点，以建立购租并举的住房制度为主要方向，深化住房制度改革，发展住房租赁市场，优化房地产供给结构，积极化解房地产库存。开展降本增效专项行动，降低制度性交易成本、人工成本、财务成本、物流成本，落实国家税费减免政策，清理规范中介服务，清理和废除妨碍公平竞争的各种规定。支持企业技术改造和设备更新，鼓励企业开发适销对路产品，提高产品质量，扩大有效和中高端供给，增强供给结构对需求变化的适应性和灵活性，提高全要素生产率。促进供给与需求有效对接、投资与消费更好结合，培育发展新动力。发挥消费对增长的基础作用。积极培育消费新热点，增加中高端消费。加强城市共同配送体系标准化、信息化建设。新建、改造农贸市场15处。完善产销对接公共服务平台，促进城乡消费融合发展。扩大消费信贷，加强市场监管，优化消费环境。发挥投资对增长的关键作用。优化投资结构，增加有效投资，抓好总投资6500亿元左右的190个市级重点建设项目，完成投资1500亿元以上。加快一汽大众华东生产基地、惠普全球大数据中心、万达东方影都、中铁世界博览城等重大项目建设，力争比亚迪新能源汽车、海晶化工等60个项目竣工投产。放宽基础设施和公用事业领域市场准入，推广政府与社会资本合作模式，激发社会投资活力。

　　大力实施创新驱动发展战略。坚持以人才和科技创新为核心，以产业创新为主导，以提质增效和可持续发展为主线，加快建设国家东部沿海重要的创新中心。把人才作为创新的第一资源，深入实施百万人才集聚行动，启动高层次人才支撑计划，组建“一带一路”国际人才智力合作联盟，推进海外高层次人才在青开展离岸创新创业。推进孵化器、软件园、人才公寓三个千万平米工程建设，搭建社会化全媒体创投平台，打造10个创业街区、100家众创空间，鼓励发展众创、众包、众扶、众筹等新模式，完善创新组织体系，营造良好创新生态，建设创新之城、创业之都、创客之岛。瞄准高端前沿，推进海洋科技、高速列车、石墨烯等十大科技创新中心建设，加快中船重工青岛海洋装备研究院、阿斯图中俄科技园等创新平台建设。强化企业创新主体地位和主导作用，深入实施中小企业“千帆计划”，加快培育具有国际竞争力的创新型领军企业。组织实施一批技术创新重点项目，支持企业承担国家重大科技项目，突破关键核心技术。完善科技成果确权、评估、交易和收益分享机制，促进科技成果转化。建立财政科技投入联动机制，支持天使投资、风险投资发展，促进科技与金融深度结合。推进知识产权综合管理改革，强化知识产权运用和保护。坚持蓝色高端新兴导向，以科技创新促进产业创新，推动产业向中高端迈进，加快向创新驱动发展转型。实施“互联网+”发展规划，推动互联网、大数据、云计算、物联网与三次产业深度融合，促进产业组织、商业模式、供应链创新。发展海尔、红领等互联网工业企业集群，打造国内重要的互联网工业强市和智能制造基地。推动300个工业转型升级项目，做优做强十大新型工业千亿级产业。滚动推进150个新兴产业重点项目，培育壮大机器人、三维（3D）打印、虚拟现实等十大战略性新兴产业，加快提高先进制造业和高技术制造业比重。深化质量强市和品牌建设，实施“标准化+”战略，打造具有国际水平的“青岛标准”。

　　促进服务业优质高效发展。坚持以金融、信息服务为核心，以全面提升现代服务业高端化能力建设为主导，以业态模式创新、形成辐射带动竞争优势为主线，加快建设国内重要的区域性服务中心。推进十个服务业千万平米工程，发展十大现代服务业，推动生产性服务业向专业化和价值链高端延伸，推动生活性服务业向高品质转变。发展金融业，加快引进和培育法人金融机构和财富管理机构，发展新型金融业态，健全多层次资本市场体系，加快提升面向国际的财富管理服务能力。发展科技服务业，构建创业孵化、技术转移、知识产权等九大服务体系，建设高水平科技信息公共服务平台，突破发展大数据、云计算、移动互联网等新型信息技术服务，加快提升现代科技服务能力。发展现代物流业，加快建设新机场、邮轮母港、董家口港，建设欧亚大通道多式联运枢纽、中国北方水产品交易和冷链物流基地，增开直达欧美重要门户城市国际航线，加快提升国际国内统筹、海陆统筹的客货运枢纽服务能力。发展现代旅游业，加快旅游基础设施和旅游休闲度假项目建设，丰富旅游产品供给，促进旅游业提质增效、全域发展，打造国际化旅游目的地城市，加快提升旅游服务能力。发展现代商贸业，打造时尚消费聚集区，推进免税购物和品牌购物中心建设，发展连锁经营、电子商务，完善现代商贸流通服务体系，加快提升消费服务能力。发展高端商务服务业，推动广告、咨询、评估、法律、仲裁、会计、审计等服务业聚集发展，提升中介服务专业化国际化水平，加快提升国际性商务服务能力。发展文化创意产业，加快文化产业园区和重点项目建设，做大影视文化、动漫游戏、出版发行、数字传媒等产业，推动文化与相关产业融合发展，促进文化产品数字化、网络化，加快提升文化创意服务能力。规模化引进高端、国际、特色教育优质资源，鼓励发展高水平、多元化技能教育和再教育，支持促进教育产业发展，加快提升教育服务能力。发展体育产业，打响奥帆品牌，加快体育产业结构调整，培育体育健身、赛事开发、体育用品制造、体育培训等产业，扩大体育产品和服务供给，加快提升体育服务能力。发展健康养老服务业，推进疗养康复、养生保健、养老服务、健康管理等产业发展，加快崂山湾国际生态健康城建设，加快提升健康养老服务能力。发展会展业，加快建设西海岸国际会展中心、红岛国际会展中心，发展会展新业态，繁荣会展市场，打造一批知名品牌展会，加快提升会展服务能力。发展总部经济，大力引进知名企业总部，做大做强现有企业总部，培育中小企业总部集群，促进总部经济聚集发展，加快提升全球化总部经济服务能力。

　　增创海洋经济新优势。坚持以海洋科技创新为制高点，以实施“一带一路”战略为主导，以海洋高端新兴产业发展为主线，加快建设国际先进的海洋发展中心。全面实施“海洋+”发展规划，推进蓝色跨越，建设海洋强市。瞄准海洋科技前沿，发挥海洋科学与技术国家实验室、国家深海基地等高端平台的支撑作用，增强协同创新和集成创新能力，努力在海洋基础研究和关键核心技术领域取得突破，抢占海洋科技制高点。加快海洋特色产业园建设，重点推进总投资1200亿元的140个蓝色经济项目，壮大海洋生物、海洋新材料、海水综合利用等新兴产业，培育海洋信息服务、海洋文化体验、海洋休闲旅游等新兴业态。依托“一区一带一园”，集聚创新要素，整合创新资源，提高海洋科技研发、成果孵化和产业化能力，打造中国蓝谷。西海岸新区培育壮大港口物流、船舶和海工装备等海洋优势产业集群，打造海洋高端产业集聚区。红岛经济区加快建设蓝色生物医药孵化中心、国家海洋技术转移中心和国家大学科技园等创新平台，打造海洋新兴产业孵化区。发展深海探测开发装备产业，建设深远海开发综合保障基地。加快东亚海洋合作平台建设，深化与“一带一路”沿线国家海洋领域的交流合作，提升海洋经济国际化水平。

　　大力推进农业现代化。以建设国家都市型现代农业示范区为抓手，加快发展十大现代农业，推进农业发展方式转变。实施藏粮于地、藏粮于技战略，启动建设100万亩粮食生产功能区、100万亩高效设施农业生产功能区、100万亩特色果茶生产基地。加快发展现代种业。推进畜禽、水产规模化健康养殖和集约化经营，发展高端特色现代畜牧业和水产业。加强农田水利设施建设，发展农田节水灌溉。推进农业全面全程机械化。加大农业科技创新和推广力度。培育新型农业经营主体和服务主体，发展多种形式适度规模经营。扶持发展农产品品牌。实施“互联网+”现代农业行动计划，推动农产品加工业转型升级，加快发展休闲农业和乡村旅游业，促进农村一二三产业融合发展。深化农村集体产权制度改革。完善“四权”抵押贷款政策，扩大农业保险覆盖面，推动金融资源更多向农村倾斜。

　　（二）坚持协调发展，着力形成平衡发展结构。坚持区域协同、城乡一体、物质文明精神文明并重、经济建设国防建设融合，在协调发展中拓宽发展空间，在加强薄弱领域中增强发展后劲，在补齐短板中促进发展平衡。

　　推动区域协调发展。深化实施全域统筹、三城联动、轴带展开、生态间隔、组团发展的城市空间发展战略，坚持一张蓝图干到底。推进“多规合一”，全面开展城市设计，科学谋划城市“成长坐标”。全面对接国际城市标准，加快基础设施建设，提升服务功能，建设现代化海湾型都市区。加快老城区改造，重点突破市北、李沧，加快邮轮母港片区建设，集聚邮轮旅游和特色金融等高端服务业，打造邮轮母港国际金贸中心和邮轮经济试验区；推进楼山工业区转型发展，建设世园生态新区。加快市南时尚消费中心和崂山金家岭财富金融聚集区建设，做大做强城阳总部经济和轨道交通产业，彰显区域功能特色。蓝谷核心区加快创新平台建设，完善综合服务功能，打造滨海生态科技新城。西海岸新区加快实施三年行动方案，创新体制机制，促进转型发展，增强综合实力，在带动区域协同发展中发挥更大作用。红岛经济区壮大新一代信息技术、高端智能制造等产业规模，加快建设市民健康中心、残疾人康复中心、科技馆和青岛中学，开工建设市民健身中心，建设科技人文生态新城。加快北部崛起，推动资金、基础设施、高端制造等向北部倾斜。平度重点推进国家中小城市综合改革试点工作，规划建设青岛北部医疗中心、文博中心、体育中心和临空经济北区，打造现代化中等城市；莱西重点推进新能源汽车和石墨烯产业基地建设，加快建设半岛交通物流中心，积极发展休闲特色产业；胶州加快临空经济示范区和国际空港城建设；即墨加快汽车产业基地、商贸名城建设，促进县域经济特色化发展。

　　推动城乡协调发展。促进基础设施建设城乡一体化。推进新机场和机场综合交通体系建设。开工建设地铁4号线、8号线，加快地铁2号线、1号线和蓝谷、西海岸城际快线建设，地铁3号线全线开通运营。推进济青高铁、青连铁路、青平城际、潍莱高铁、平度至新机场快速路建设。落实城市地下空间利用总体规划，加大地下空间资源开发利用力度。统筹规划城镇地下管线，加快建设地下综合管廊。加强信息基础设施建设，构建普惠性信息网络，打造智慧青岛。推进重点输变电工程和充换电网络建设，完成3000个村低压电网改造。市区新增集中供热面积400万平方米。实施水源、调水、中小河道治理等工程。推进以人为核心的新型城镇化。做好国家新型城镇化试点工作。推进小城市培育试点和省级示范镇建设，打造一批特色小镇。推进新型农村社区和特色产业园区建设，完善提升200个社区服务中心。加强传统村落民居和历史文化名村名镇保护。加强农村人居环境整治，改造农村危房6000户，改造农村卫生厕所30万座，建设美丽宜居乡村。深化户籍制度改革，探索建立居住证与教育、医疗、保障房等基本公共服务供给衔接制度，提高户籍人口城镇化率。

　　推动物质文明和精神文明协调发展。坚持用中国梦和社会主义核心价值观凝聚共识、汇聚力量。加强思想道德建设，弘扬中华传统美德，倡导志愿服务，发展公益事业，传播正能量，使向上向善、诚信互助的社会风尚更加浓厚。深化新一轮全国文明城市创建，完善群众性精神文明创建活动长效机制，提升城市文明程度。加大文化环保城市建设力度。完善公共文化服务体系，推进市文化中心、市博物馆改造等项目建设，加强基层综合文化服务中心建设，更多向社会力量购买公共文化服务。打造“五王大赛”文化惠民活动品牌。建立完善影视产业发展服务体系，打造影视文化高地。加快国际版权交易中心、国家广告产业园建设。积极申办亚洲艺术节、国际戏剧文化节。加强历史文化名城保护，重视历史优秀建筑开发利用，打造海滨历史文化长廊。搭建国际文化交流平台，推进东亚文化之都城市联盟建设。加强全民健身设施建设。积极发展校园足球、社会足球、职业足球，建设以笼式足球场为主的多功能运动场地100处。办好克利伯环球帆船赛、世界杯帆船赛、亚洲大众体操节等赛事。加快建设第24届省运会比赛场馆。做好民族宗教、侨务、对台工作，推动统计、档案、史志、仲裁事业发展，提高人防、气象、防震减灾工作水平。

　　推动军民融合深度发展。积极开展军地共商、产业共融、科技共兴、设施共建、后勤共保，创新军民深度融合体制机制，创建古镇口军民融合创新示范区，探索具有青岛特色的军民融合深度发展新模式。服务国防和军队改革，推进国防教育和民兵预备役建设，做好双拥共建工作，密切军政军民关系。

　　（三）坚持绿色发展，着力改善生态环境。坚持生态兴市、绿色惠民，加大资源节约和环境保护力度，推行绿色发展方式和生活方式，打造美丽青岛、宜居城市、幸福家园。

　　促进人与自然和谐共生。严格实施城市规划“七线”管控和生态环境空间规划，促进形成更加合理的城市空间结构。实施山水林田湖生态保护和修复工程，构建多层次、网络化的生态间隔体系。落实主体功能区规划，加强对崂山等风景名胜区、自然保护区、海洋生态保护区，以及湿地、水源地等特殊生态功能区的保护。开展国土绿化和城市绿荫行动，实施生态提升“十大工程”。规划建设青岛北部生态屏障区。推进小麦岛生态修复和浮山综合治理。

　　加大环境治理力度。编制空气质量达标规划，落实大气污染防治计划。实施多污染物协同控制，抓好燃煤废气、机动车尾气、工业有机废气和扬尘治理，完成10台燃煤机组（锅炉）超低排放改造，力争市区空气细颗粒物（PM2.5）年均浓度下降5%。编制胶州湾保护总体规划，开展蓝色海湾治理行动，创建胶州湾国家级海洋公园。推进河流污染整治，墨水河等主要河流水质明显改善。强化土壤污染防治，开展污染场地治理和修复试点示范。加大农业面源污染防治力度，推进种养业废弃物资源化利用、无害化处置。

　　推动低碳循环发展。开展国家低碳城市试点。支持绿色清洁生产，对传统制造业实施绿色改造，鼓励新能源汽车等节能环保产业加快发展。开展重点用能单位节能低碳行动，实施重点产业能效提升计划。发展循环经济，推进“城市矿产”、建筑废弃物综合利用等试点工作，打造国家循环经济示范城市。加快绿色新城区建设，完成既有建筑节能改造200万平方米。因地制宜发展风能、太阳能、海洋能、生物质能等清洁能源。

　　高效节约利用资源。强化约束性指标管理，开展能源和水资源消耗、建设用地等总量和强度双控行动，实施全民节能行动计划。推广海水淡化，建设节水型社会。规划建设海绵城市。实施耕地质量提升计划，全面划定永久基本农田，提高土地利用效率。开展绿色生活行动，倡导绿色低碳消费。

　　建立生态文明制度体系。加大对生态保护区和农产品主产区转移支付力度，创新生态补偿机制。建立陆海统筹污染防治机制。落实污染物排放许可制度，开展排污权有偿使用和交易试点。探索建立用水权交易市场。推进碳排放权交易先行先试。完善企业环境行为评价约束制度，严厉查处环境违法违规行为。完善环境监测预警机制，提高环境风险防控和突发环境事件应急能力。

　　（四）坚持开放发展，着力再造竞争新优势。坚持改革不停顿、开放不止步，打好改革攻坚战，提升城市国际化水平，努力在新一轮改革开放中走在前列。

　　构建开放型经济新体制。制定国际城市战略实施纲要及年度行动计划。复制推广上海等自贸试验区经验，推行准入前国民待遇加负面清单管理制度。积极申办自由贸易试验区。推进出口加工区转型升级为综合保税区。加快建设跨境电子商务综合试验区，推进青岛邮政跨境电商产业园、胶州湾国际物流园建设，积极申建国家铁路临时开放口岸。推动即墨开发区升级为国家级经济技术开发区。发挥胶州多式联运海关监管中心优势，打造综合贸易枢纽。争取董家口一类口岸开放，力争开通青满欧铁路货运班列，创新多式联运新模式，打造“一带一路”国际大通道。推进公共检测服务平台建设，加快电子口岸平台和国际贸易“单一窗口”建设，打造高效口岸通关模式。

　　提高开放型经济发展水平。深度融入国家“一带一路”战略，扩大与沿线国家和地区的交流与合作，提升“引进来”和“走出去”的质量效益。创新利用外资方式，鼓励外资以参股、并购等方式参与企业兼并重组。制定实施青岛与世界500强和全球行业领军企业合作行动计划。扩大金融、教育、养老、医疗、文化等服务领域开放。推进货物贸易向优进优出转变，扩大机电产品和高新技术产品出口。促进服务贸易实现跨越发展，创建国家服务外包示范城市。支持外贸综合服务平台建设。培育一批国家、省级外贸生产基地和外贸品牌小镇。优化进口结构，建设进口商品促进中心和分销中心，推动内外贸融合发展。加快中德生态园、欧亚经贸合作产业园和中韩、中日创新产业园建设。推动境外经贸合作园区建设。发挥中美、中英地方经贸合作重点城市双向开放作用，深化与国际友好城市、友好合作关系城市的全方位交流与合作。加大对口帮扶力度，加强区域经济合作。

　　深化重要领域和关键环节改革。大力推进简政放权、放管结合、优化服务改革，进一步削减、下放行政审批事项，全面清理前置审批事项，优化行政审批流程，强化事中事后监管。加快公共资源交易改革，实现服务、管理与监督相分离，建立健全统一规范的公共资源交易平台。全面开展区市综合行政执法改革。完成国内贸易流通体制改革发展综合试点任务。推进旅游业改革创新先行区建设，完善旅游管理体制，做大做强市场主体。深化财税体制改革，实行跨年度预算平衡和中期财政规划管理。改革财政资金运作方式，壮大股权投资引导基金规模。加强政府性债务管理。加快财富管理金融综合改革试验区建设，积极申请第二批创新试点政策。组建金融衍生品交易中心等要素市场，完善财富管理基础业态。支持地方法人金融机构综合经营试点，鼓励大企业发起设立或参与组建金融机构。发挥蓝海股权交易中心作用，引导企业通过上市、发行债券、私募股权等渠道直接融资。完善金融风险处置应急机制，严厉打击非法集资行为，积极防范和化解金融风险，进一步优化金融生态。

　　激发各类市场主体活力。深化商事制度改革。鼓励非国有资本参与国有企业改革，稳妥推进国有企业混合所有制改革试点。加大国有企业职业经理人市场化选聘力度。加强国有资产监管，提高国有资本运营效率。支持非公有制经济健康发展，激发非公有制经济活力和创造力。弘扬企业家精神。依法保护公民和各类市场主体财产权和创新收益。加快价格改革步伐，建立大宗商品价格指数体系。

　　（五）坚持共享发展，着力增进人民福祉。坚持以人民为中心的发展思想，解决好事关群众切身利益的问题，努力提高群众对居住环境、看病就医、文化生活等方面的满意度。重点办好10件36项市办实事。

　　提高教育质量。推进教育综合改革，增强学生的社会责任感、创新精神和实践能力。重视学前教育普惠发展，新建改建幼儿园60所。推动义务教育均衡发展，鼓励优质中小学跨区域合作办学，改造100所农村义务教育薄弱学校。落实乡村教师支持计划。统筹解决城镇普通中小学大班额问题，95%的中小学达到市现代化学校标准，确保5所寄宿制普通高中投入使用。建设中小学标准化食堂200所。优化职业院校布局，推动中职、高职、本科一体化人才培养。加快幼儿师专建设和省轻工工程学校扩建。山东大学青岛校区正式招生。加快建设中国科学院科教融合基地、中国科学院大学海洋学院和对外经贸大学青岛研究院，推进青岛农业大学平度创新创业基地建设。健全终身教育体系。提升特殊教育发展水平。支持和规范民办教育发展。建设教育管理公共服务平台，85%的中小学建成智慧校园。加强教育国际交流与合作，积极引进国内外优质高等教育资源。承办全国第五届中小学生艺术展演。

　　促进就业创业。落实就业优先战略，加强经济政策与就业政策配套衔接，完善重点建设项目与扩大就业联动机制。促进以高校毕业生为重点的青年就业和城镇困难人员、残疾人就业，加强对灵活就业和新就业形态的扶持，城镇新增就业30万人。推进大众创业和返乡创业工程，实施高校毕业生创业引领计划，扶持创业3万人。推行终身职业技能培训制度。完成职业农民技能证书培训1.5万人。

　　完善社会保障制度。落实全民参保计划，抓好扩面征缴工作。建立完善补充医疗保险制度，完善城乡居民大病保险制度。提高企业退休人员基本养老金标准。提高城乡低保标准，实施新的城乡困难居民医疗救助制度。支持慈善事业发展，完善大救助平台，让困难群众及时得到救助。完善重度残疾人护理补贴和困难残疾人生活补贴制度，加快残疾人小康进程。建设30处社区小型养老机构，支持社会力量提供养老服务。新增养老床位4000张。完善长期护理保险制度，为60周岁以上老年人购买意外伤害保险。实行60－64周岁老年人半价乘坐公共交通工具。发展临终关怀事业。建立健全农村留守儿童和妇女、老人、残疾人关爱服务体系。扩大住房租赁补贴保障范围，完成住房保障8000套（户）。加快城中村改造。启动棚户区改造6万户，其中主城区2.4万户。

　　提前完成脱贫攻坚任务。健全精准识别机制，全面核查贫困村和贫困人口，做到精准到户、精准到人。落实脱贫工作责任制，坚持专项扶贫、行业扶贫和社会扶贫联动，通过培育特色产业、就业创业、兜底保障、教育保障、医疗保障、交通保障等综合措施，市定标准内3万农村贫困人口全部脱贫。

　　推进健康青岛建设。加快城市公立医院综合改革，巩固县级公立医院改革成果。坚持医药分开，实行分级诊疗，巩固基本药物制度。推进市公共卫生中心、第八人民医院、眼科医院新院区等项目建设。实施“互联网+”医疗健康行动计划，建设数字化医院。争取建设国家级中医药健康服务业综合示范区。加强全科医生、家庭医生和乡村医生队伍建设，促进医疗资源向基层和农村流动。鼓励社会办医。完善医疗纠纷调解机制，构建和谐医患关系。落实一对夫妇可生育两个孩子政策。探索建立计划生育特殊家庭保障体系。

　　强化重点领域专项治理。开展消费市场秩序专项整治，加大餐饮、住宿、交通、旅游等服务领域执法力度，以零容忍的态度严厉打击侵害消费者权益行为。落实安全生产“党政同责、一岗双责、失职追责”要求，强化生产经营单位主体责任。实施化工产业安全环保转型升级工程，提升安全生产、环境治理和节能降耗水平。深入实施“食安青岛”行动，抓好食品安全源头治理，开展食品安全定性定量检测6.5万批次、农产品质量快速检测20万批次，创建国家食品安全城市、农产品质量安全市。深化城市管理执法体制改革，推进管理重心下移，提升智慧化精细化管理水平。下气力治理乱停车、露天烧烤、占路经营、违章建筑等城市顽疾，下决心规范机场、车站周边秩序，巩固卫生城市创建成果。坚持公交优先，实施公共交通换乘优惠。推进公共停车场建设，改造道路交通拥堵节点，新建改造道路交通安全设施450处。实施公路安全生命防护工程，完成农村公路隐患整治1000公里。加强城乡公共消防设施建设。建成公共信用信息平台，整合共享公共信用信息，完善社会信用体系。加强信访法治化建设，开展信访积案攻坚化解年活动。加强普法教育。深化平安青岛建设，构建社会治安立体防控体系,健全社会矛盾排查预警和调处化解综合机制，严密防范、依法惩治违法犯罪活动。开展基层应急能力提升年活动，推进应急管理标准化建设，加强市民应急培训和中小学生应急体验教育。

　　四、加强政府自身建设

　　深入贯彻落实法治政府建设实施纲要，依法全面履行政府职能，不断提高政府的公信力和执行力，加快建设法治政府、创新政府、廉洁政府和服务型政府。

　　（一）坚持依法行政。在市委的坚强领导下，自觉接受人大法律监督、工作监督和政协民主监督，坚决执行人大及其常委会的决议、决定，落实重大事项向人大及其常委会报告、向政协通报制度，认真听取各民主党派、工商联、无党派人士和人民团体的意见。扎实做好建议提案办理工作，切实提高办理工作落实率和代表委员满意率。推进行政决策科学化、民主化、法治化，提高决策质量和水平。深入贯彻《立法法》，积极推动重点领域立法，更好发挥法治的引领和规范作用。全面落实行政执法责任制，规范行政执法行为。健全法律顾问制度，依法开展行政调解、行政裁决工作。加强政府工作人员法治教育，自觉做到尊法学法守法用法，自觉运用法治思维和法治方式开展工作，营造良好的法治环境。

　　（二）深化政务公开。推进政府预算和决算向社会公开，所有财政拨款的“三公”经费逐步公开。推进公共资源配置、重大建设项目批准和实施、社会公益事业建设等重点领域信息公开。推行“互联网+”政务服务，推进行政权力网上规范透明运行。启动政务大数据中心建设，扩大政府数据向社会开放。规范政府新闻发布，加强政策解读，积极及时回应群众关注的热点难点问题。继续开展“三民”活动，整合政务服务热线，发挥网络在线问政、行风在线等互动平台作用，主动自觉地接受舆论监督和公众监督。

　　（三）改进政府作风。巩固扩大“三严三实”专题教育成果，抓好“两学一做”学习教育。加强干部队伍教育培训，提高综合素质和专业素养。持续寻标对标达标夺标创标，不断加速提升创新增效落实。继续开展进现场解难题促转型稳增长抓落实工作推进活动，深入开展走访千家企业、走进千个项目现场、走谈千个项目活动。落实联系服务代表委员、基层单位等制度。建立健全政策落实的责任机制、奖勤罚懒的激励机制和常态化的督查问责机制，把先行先试与明知故犯、探索实践与我行我素、无意过失与违纪违法区分开来，着力解决能力不足而“不能为”、动力不足而“不想为”、担当不足而“不敢为”的问题，推动形成想作为、敢作为、善作为的良好风尚。

　　（四）建设廉洁政府。坚持纪严于法、纪在法前，认真贯彻落实《中国共产党廉洁自律准则》《中国共产党纪律处分条例》。严格按照党的原则、纪律、规矩办事，不滥用权力，不违纪违法。加强对维护党章、执行党的路线方针政策和决议情况的监督检查，确保政令畅通。持之以恒落实中央八项规定精神，紧盯重要节点，畅通监督渠道，创新监督方式，严肃整治顶风违纪行为，严防“四风”现象反弹回潮。坚持“一岗双责”，落实从严治党主体责任，严格责任追究。加强对权力运行的制约监督，建立健全权力清单、责任清单和负面清单制度，扎紧织密制度的笼子。探索推进“互联网+”廉政，深化全程电子监察。加强对重大决策部署落实情况的监督检查和跟踪审计，实现对公共资金、国有资产、国有资源和领导干部经济责任履行情况审计监督的全覆盖。坚决查处违纪违法案件，加大对发生在群众身边腐败问题的查处力度，积极构建不敢腐、不能腐、不想腐的有效机制，进一步营造风清气正、务实为民、干事创业的良好环境。

　　各位代表，新的一年充满新的希望，新的征程承载新的梦想。我们一定牢固树立政治意识、大局意识、核心意识、看齐意识，更加紧密地团结在以习近平同志为总书记的党中央周围，在中共青岛市委的坚强领导下，凝心聚力，奋力进取，为加快建设宜居幸福的现代化国际城市作出更大贡献！
